# Sympathy towards people infected with COVID-19 mediates relations between media use and death anxiety

**DOI:** 10.3389/fpsyt.2025.1570747

**Published:** 2025-04-08

**Authors:** Miao Chao, Jie Liu, Dini Xue, Bin Zhang, Brian J. Hall

**Affiliations:** ^1^ Key Research Base of Humanities and Social Sciences of the Ministry of Education, Academy of Psychology and Behavior, Tianjin Normal University, Tianjin, China; ^2^ Faculty of Psychology, Tianjin Normal University, Tianjin, China; ^3^ Tianjin Key Laboratory of Student Mental Health and Intelligence Assessment, Tianjin, China; ^4^ School of Global Public Health, New York University Shanghai, Shanghai, China

**Keywords:** media use, sympathy, death anxiety, COVID-19, longitudinal

## Abstract

The COVID-19 pandemic threatened mental health. This study examined the longitudinal associations among pandemic-related media use, sympathy for people infected with COVID-19 (PIWC), and death anxiety. 132 Chinese adults completed measures three times, one week apart during the initial phase of COVID. The results showed that media use, sympathy, and death anxiety decreased significantly over the first month of the outbreak. Pandemic-related media use predicted increased future sympathy for PIWC, and sympathy predicted increased future death anxiety. The study identified the short-term effects of media use on sympathy and death anxiety, and suggests that reduced media exposure may be beneficial.

## Introduction

The COVID-19 pandemic continues to reveal long-term effects on public health and psychological adaptation in the post-crisis era. While quarantine and social distancing measures have transitioned from emergency protocols to routine public health strategies, their psychological consequences demand sustained attention. Contemporary media environments maintain persistent mortality reminders through residual pandemic narratives - from archival infection data to enduring visual symbols of personal protection equipment. Understanding how media coverage impacts death anxiety is critical to limit the negative effect of the pandemic.

Fear of death has been a pervasive theme in human history, and it has recently been proposed as a transdiagnostic factor, which might be a basic fear at the core of a range of mental disorders, including hypochondriasis, panic disorder, and anxiety and depressive disorders (1, 2). In the pandemic situation with ubiquitous salience of mortality, death anxiety may be easily triggered, increasing psychological distress (3, 4). Death anxiety may drive a significant amount of mental health problems during the pandemic. For example, a study on 1210 Chinese showed that low estimates of one’s survival from COVID-19 predicted higher levels of stress and depression in the pandemic (5). In addition, death anxiety significantly predicts coronavirus anxiety beyond sociodemographic variables in an European sample (6).

During the pandemic, the public is highly dependent on accurate and up-to-date information on the media to make informed decisions (7). However, psychological distress resulting from repeated media exposure began to emerge. Associations between media exposure and mental health problems during the COVID-19 pandemic have been reported in many studies, such as negative affect, death anxiety, sleeping problems, depression, anxiety, and stress (8–11).

Uncertainty reduction theory posits that individuals seek information to mitigate distress caused by ambiguous threats (12). During the pandemic, media use served as a critical tool for reducing uncertainty, which may explain initial high levels of information-seeking behavior and subsequent declines as uncertainty decreased. As a relevant psychological consequence of the pandemic, the relationship between death anxiety and media exposure has received less attention.

Generally, attitudes about death can be affected by media exposure. For instance, media representations of group deaths elevate both overt and covert death fears of college students (13). Media use related to the pandemic may also impact one’s death attitude. On one hand, media exposure to various COVID-related contents was found to be positively associated with death anxiety (9). On the other hand, feeling informed might have a buffering effect on people’s emotional state. The perception of being informed about various issues of the COVID-19 pandemic was consistently negatively correlated with current virus anxiety, health anxiety, and cyberchondria (14). Thus, the role of media use on death anxiety in the pandemic needs further examination.

Moreover, it may also be that individuals with more death anxiety purposefully sought out more media information on the pandemic, helping them diffuse their own fears via promoting a sense of control. Therefore, one aim of the present study was to examine whether pandemic-related media use was related to death anxiety in the COVID-19 pandemic using longitudinal design.

In the pandemic, sympathy is a common emotion experienced by most people. Sympathy is defined as feelings of sorrow or concern for another’s welfare (15). People tend to express sympathy towards victims of disasters (16, 17). Loewenstein and Small (18) proposed that whether one is in the same state as the victim, vicarious experiences, proximity, similarity, vividness, and newness may affect the strength of sympathy. In the pandemic, all people are in the similar situation of possibly contracting COVID-19, and media coverage provides vivid reports of the pandemic. Moreover, although sympathy is an other-regarding emotion, it depends on the imagination of the emotions in the sympathizer rather than the actual emotion of others. This imagination might be based on the pandemic-related media use to a certain extent. Therefore, for those who are not infected with the virus, media use may heighten their awareness of others’ suffering and provide an opportunity to understand the negative experiences, inducing feelings of sympathy towards victims.

When witnessing others’ suffering, the observer’s reactions may include fear of personal safety to concern for others. At present, little is known about the relationship between concerns for others (i.e., sympathy) and concerns for oneself (i.e., death anxiety/fear). The current study attempted to test whether sympathy towards others and fear of one’s own death are related.

Several previous studies are instructive. On one hand, sympathy might be a responsive emotion elicited by feelings of fear. For instance, experimental studies demonstrated that fear-primed participants reported more sympathy and desire to help the protagonists than neutral-primed participants (19). Similarly, when confronted with an anxious person, if one senses signs of fear in his own body, he will engage in sympathetic resonance (20). Therefore, it is possible that death anxiety would lead to sympathy for others. On the other hand, sympathy for the victims during the pandemic might also negatively impact mental health, including elevated death anxiety. A study on frontier-line nurses found their depression usually stems from sympathy for COVID-19 patients (21). Another study on the general public showed that sympathy for the infected might affect their own death anxiety (9). In fact, literature has suggested vicarious traumatization through the media led to higher anxiety in the pandemic (22), and vicarious traumatization is typically derived from sympathy for the victims (21). In the COVID context, it is important to understand the longitudinal correlation between death anxiety and sympathy.

### The current study

In sum, although studies are abundant regarding the media use behaviors in the pandemic (7, 8, 10, 22), the previous studies seldom looked into the temporal changes of media use. In addition, despite evidence found supporting the link between media exposure, sympathy and death anxiety, few studies have examined the relationship concurrently, and little is known about the directionality of the relationship. Therefore, the current study will investigate media use behaviors, sympathy for the victims and death anxiety of Chinese citizens using a short-term longitudinal design.

Three-wave data was collected with a short interval of one week in the beginning of the pandemic from late January to February 2020 when the pandemic was drastically developed, as daily new cases increased from 1459 to 3884 and 2015 at the three timepoints we collected data (WHO). The pandemic was changing rapidly and affected the public’s risk perception, which was closely linked to information seeking behaviors and fear (23, 24). Therefore, the one-week interval was chosen to capture rapid shifts in risk perception during the outbreak’s initial phase.

The study examined three research questions: (1) the condition and change of media use behavior, sympathy and death anxiety of the general public, and (2) the longitudinal correlation of media use behavior, sympathy and death anxiety, (3) whether sympathy can act as mediator of media use and death anxiety, or whether death anxiety could mediate the relation between media use and sympathy in the pandemic.

Although the acute phase of the pandemic has subsided, understanding its psychological legacy remains important for informing public health strategies during future crises. The findings could elucidate how media exposure during emergent threats can amplify vicarious trauma, offering timeless insights into crisis communication.

## Methods

### Participants

The G*Power program was used to calculate the number of participants required for regression analysis in this study with an effect size f^2^ of.15, a significance level of.05, a statistical power of.95, a predictor number of 6, and a two-sided test, in accordance with the purpose of this study. As a result, a minimum of 89 participants were required.

We recruited 132 healthy Chinese adults through a popular social media platform using snowball sampling method. The participants completed scales 3 times from January 28, 2020, with an interval of one week through Tencent Questionnaire. Participants received no monetary incentives. Retention was achieved through personalized reminders via Tencent Questionnaire, and the platform required all items to be completed before submission, minimizing attrition. So there was no missing data.

Demographics were collected at Wave 1; otherwise, scales were the same in all waves. Among the 132 participants (with one participant’s demographic information missing), 95 (72.0%) were women and 36 (27.3%) were men, with a mean age of 27.0 years (SD = 9.2), and 73 (55.3%) were single. Six (4.5%) participants received a high school diploma, 9 (6.8%) received vocational education, 72 (54.5%) received college education, and 44 (33.3%) had graduate degree or above. Among the participants, 79 (59.8%) were students, 39 (29.5%) were employed in various industries, 5 (3.8%) were current unemployed, and 8 (6.1%) were in other occupations. Neither the respondents nor anyone they knew were reported infected with COVID-19. The participants were from 23 provinces, municipalities and autonomous regions in China. All participants were informed of the study purpose, and provided consent to participate. This study was approved by the ethical committee of [name deleted to maintain the integrity of the review process].

### Measures

#### Media use

Five questions were used to measure the time spent on pandemic-related media coverage in the past week. Participants indicated the number of total hours in the last week that they were exposed to the coverage of the pandemic via television, radio, newspapers; online news sites; or via pictures, videos; and news, or text updates on social media. An example question is “In the past week, how many hours did you spend reading online news about the pandemic?”. The five items were summed to create a media use score.

#### Sympathy

Two items were used to measure sympathy for those affected by the pandemic. The two items were “I feel sorry for others who are infected with COVID-19” and a reverse-coded item “I don’t tend to have feelings of sorrow or concern when I see other people get infected”. The responses were rated on a 5-point scale (1 = strongly disagree; 5 = strongly agree). The sum score of the two items ranging from 2 to 10 was used. A higher score indicates a higher level of sympathy. The Cronbach’s alpha values were 0.59, 0.50, and 0.62 at T1, T2, and T3, respectively.

#### Death anxiety

Four items adapted from Templer Death Anxiety Scale (25) were used to fit the pandemic context. The four items were “I am afraid of dying from the COVID”; “My heart beats faster hearing people talk about the death caused by the pandemic”; “The thought of contracting the COVID virus bothers me” and “The subject of COVID-19 causing mortality troubles me greatly”. Items were scored on a 5-point scale, from 1 = strongly disagree to 5 = strongly agree. The sum score of the four items ranging from 4 to 20 was used. The Cronbach’s alpha values were 0.75, 0.79, and 0.80 at T1, T2, and T3, respectively.

### Data analysis

Descriptive statistics were conducted with IBM SPSS 25.0. Relationships among the variables were examined using correlation analysis. Paired samples T-tests were conducted to examine changes of variables from T1-T3. Structural equation modeling (SEM) was used to conduct three-wave cross-lagged analyses using Mplus 8.0 to examine the reciprocal relations among media exposure, sympathy and death anxiety. Acceptable model fit is suggested by a χ^2^/df between 2.0 and 5.0, a comparative fit index (CFI) at or exceeding 0.95, a root mean square error of approximation (RMSEA) at or below 0.1, and a standardized root mean square residual (SRMR) at or below 0.08.

## Results

To assess potential common method variance in this study, Harman’s single-factor test was performed as the data were collected through self-reported questionnaires. The exploratory factor analysis revealed 3 factors with eigenvalues exceeding 1. Notably, the first factor accounted for 37.49% of the total variance, which is below the recommended threshold of 40%, suggesting minimal common method bias.

Descriptive statistics and the correlation matrix are presented in **Table 1**. Longitudinally, T1 media use was significantly correlated with media use and sympathy at T2 (0.49 and 0.18, respectively) and T3 (0.46 and 0.19, respectively); T1 sympathy was significantly correlated with sympathy and death anxiety at T2 (0.54 and 0.30, respectively) and T3 (0.61 and 0.21, respectively); and T1 death anxiety was significantly correlated with death anxiety at T2 and T3 (0.69 and 0.71, respectively). Cross-sectionally, the three variables were not correlated at T1. At T2 and T3, media use was correlated with sympathy (0.19 and 0.30, respectively), and sympathy was correlated with death anxiety (0.30 and 0.34, respectively).

**Table 1:** Descriptive statistics results and correlation matrix.

Variables	M ± SD	1	2	3	4	5	6	7	8	9
T1										
**1** Media use	13.67 ± 4.43	1								
**2** Sympathy	8.39 ± 1.51	0.08	1							
**3** Death anxiety	12.88 ± 3.53	0.02	0.15	1						
T2										
**4** Media use	10.43 ± 3.53	0.49^**^	0.08	-0.03	1					
**5** Sympathy	8.20 ± 1.34	0.18^*^	0.54^**^	0.19^*^	0.19^*^	1				
**6** Death anxiety	12.28 ± 3.63	0.07	0.30^**^	0.69^**^	0.07	0.30^**^	1			
T3										
**7** Media use	9.92 ± 3.48	0.46^**^	0.17	0.01	0.80^**^	0.25^**^	0.14	1		
**8** Sympathy	8.08 ± 1.41	0.19^*^	0.61^**^	0.15	0.26^**^	0.69^**^	0.32^**^	0.30^**^	1	
**9** Death anxiety	12.21 ± 3.44	0.09	0.21^*^	0.71^**^	0.10	0.34^**^	0.75^**^	0.16	0.34^**^	1

T1, Time 1, T2, Time 2, T3, Time 3, *p<0.05, **p<0.01.

**Figure 1:**
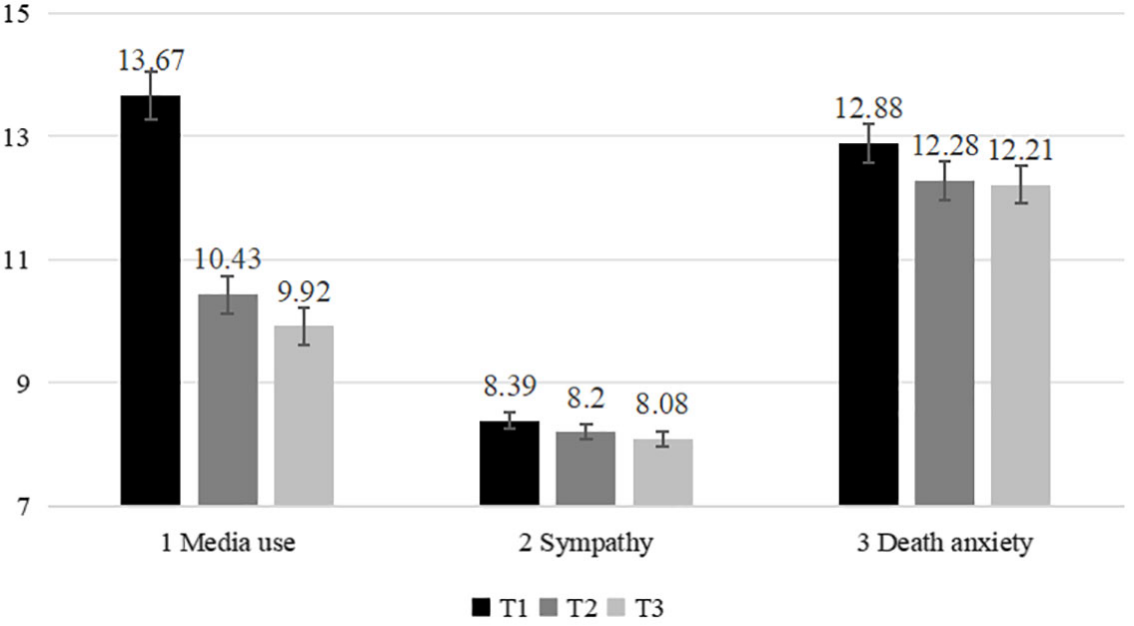
The trajectories of all the variables from T1 to T3. T1, Time 1, T2, Time 2, T3, Time 3. Error bars represent 95% confidence intervals.

The trajectories of media use, sympathy and death anxiety were presented in **Figure 1**. Generally, all the variables displayed a decrease from T1 to T3.

Besides, **Table 2** displayed the results of paired sample T-tests suggested that the media use decreased significantly from T1 to T2, T2 to T3, and T1 to T3. For sympathy, the decrease from T1 to T2 and T2 to T3 was not significant, but the decrease was significant from T1 to T3. Death anxiety declined significantly from T1 to T2, kept stable from T2 to T3, and the decrease was significant from T1 to T3. Similarly, the results of ANOVA with repeated measurements showed that factor effects for media use (*F* = 84.000, *p* <.001, partial *η* ^2^ = .391), death anxiety (*F* = 4.997, *p* = .008, partial *η* ^2^ = .037), and sympathy (F = 4.084, p = .020, partial *η* ^2^ = .030) were significant. Furthermore, pairwise comparisons with Bonferroni correction showed that the differences of sympathy T2-T1, sympathy T3-T2, and death anxiety T3-T2 were not significant.

**Table 2:** The results of paired sample T-test.

		Paired Differences				t	d
		M	SD	95% CI			
				Lower	Upper		
Media use	T2 - T1	-3.24	4.08	-3.95	-2.54	-9.13 ^***^	-1.60
	T3 - T2	-0.52	2.22	-0.90	-0.13	-2.67 ^**^	-0.47
	T3 - T1	-3.76	4.19	-4.48	-3.04	-10.31 ^***^	-1.81
Sympathy	T2 - T1	-0.18	1.38	-0.42	0.06	-1.51	-0.26
	T3 - T2	-0.13	1.08	-0.32	0.06	-1.37	-0.24
	T3 - T1	-0.31	1.29	-0.53	-0.09	-2.78 ^**^	-0.49
Death anxiety	T2 - T1	-0.60	2.83	-1.09	-0.11	-2.43 ^*^	-0.43
	T3 - T2	-0.07	2.50	-0.50	0.36	-0.31	-0.05
	T3 - T1	-0.67	2.66	-1.12	-0.21	-2.88 ^**^	-0.51

*p<0.05, **p<0.01, ***p<0.001.

Three cross-lagged models were established. In model 1, by testing both paths of media use -> death anxiety -> sympathy and media use -> sympathy -> death anxiety paths together, we examined whether both death anxiety and sympathy might act as mediators. In model 2, we tested the mediating role of death anxiety, that is, the path of media use -> death anxiety -> sympathy. In model 3, we tested the mediating role of sympathy, that is, media use -> sympathy -> death anxiety path. The model fit indices showed that CFI, TLI, and RMSEA indices of the three models were acceptable, and ACI and BIC were similar in all three models. Model 1 shows AIC=3219.983, BIC=3303.584, CFI=0.998, TLI=0.996, RMSEA=0.021, SRMR=0.051. Model 2 yields AIC=3236.067, BIC=3308.137, CFI=0.953, TLI=0.923, RMSEA=0.093, SRMR=0.100. Model 3 displays AIC=3239.543, BIC=3305.848, CFI=0.952, TLI=0.927, RMSEA=0.090, SRMR=0.078. However, the media use -> death anxiety -> sympathy path was not supported in both model 1 and model 2. Specifically, death anxiety could not significantly predict sympathy longitudinally (*β*_T1-T2_ = 0.041, *p* = 0.178, *β*_T2-T3_ = 0.028, *p*=0.182 in model 1; and *β*_T1-T2_ = 0.041, *p* = 0.186, *β*_T2-T3_ = 0.029, *p*=0.201 in model 2). Therefore, model 3 was supported.

**Figure 2:**
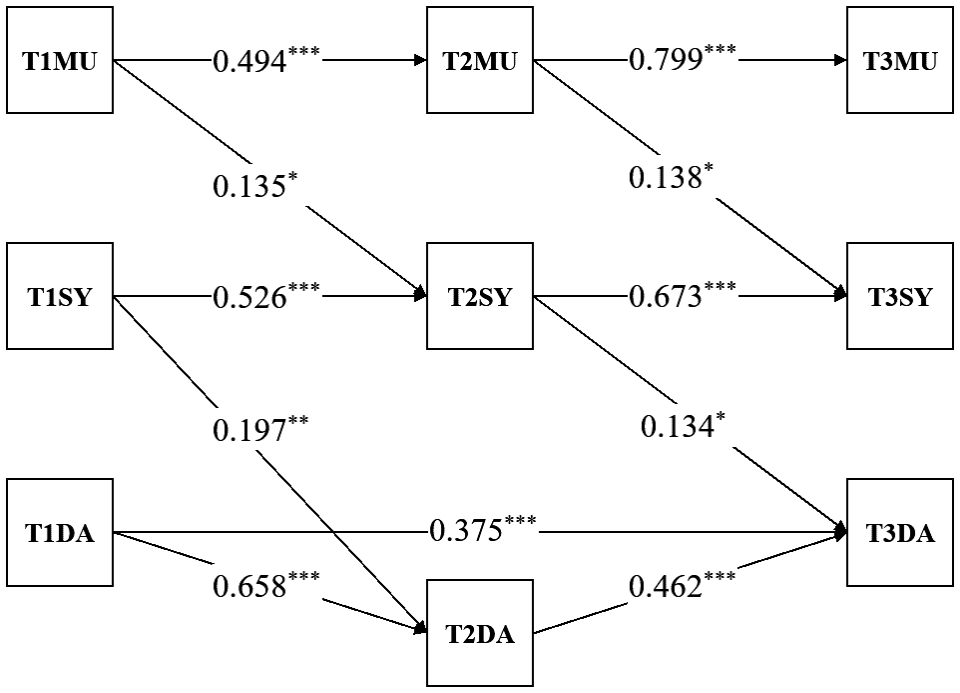
Three-wave cross-lagged model. T1MU, media use at T1; T1SY, sympathy at T1; T1DA, death anxiety at T1; T2MU, media use at T2; T2SY, sympathy at T2; T2DA, death anxiety at T2; T3MU, media use at T3; T3SY, sympathy at T3; T3DA, death anxiety at T3. *p<0.05, **p<0.01, ***p<0.001. Standardized coefficients are labeled for all paths.

The final cross-lagged model including media use, sympathy, and death anxiety, and its standardized path coefficients were shown in **Figure 2**. Firstly, auto-aggressive paths were significant for media use, sympathy, and death anxiety across time. Then it was about the mediating effect of sympathy. In the first half of the mediation, media use at T1 was a significant predictor of sympathy at T2 (β = 0.135, p < 0.05). In the second half of the mediation, sympathy at T2 was a significant predictor of death anxiety at T3 (β = 0.134, p < 0.05). It suggested there was a significant mediating pathway from media use at T1 to death anxiety at T3 through sympathy at T2. We conducted bootstrap mediation analysis with 1,000 resamples using MLR estimation. The results showed that the indirect effect of media use at T1 on death anxiety at T3 through sympathy at T2 was (*β* = 0.059, 95% *CI* [0.001, 0.121]), with the confidence interval excluding 0.

These results suggest that pandemic-related media use influence future sympathy towards the victims (over a 1-week period), and sympathy influence future death anxiety (over a 1-week period), whereas sympathy and death anxiety do not influence future media use (over a 1-week period), and death anxiety do not influence future sympathy (over a 1-week period).

## Discussion

In this short-term longitudinal study, we tracked changes in pandemic-related media use behavior, sympathy for the infected and death anxiety of healthy adult in the initial phase of the COVID-19 pandemic, and examined their longitudinal correlations. Overall, media use, sympathy and death anxiety decreased over the first month of the outbreak. Longitudinally, pandemic-related media use predicted increased future sympathy for people infected with COVID-19, and sympathy predicted increased future death anxiety. More importantly, sympathy for people infected with COVID-19 mediated the impact of media exposure on death anxiety.

The present study highlights the importance of examining the temporal changes of media use behavior for enhanced understanding of pandemic-related information seeking. Our findings suggest that media use behaviors showed particularly pronounced decrease in the first month of the pandemic. The pandemic has resulted in significant uncertainty for the public. According to the uncertainty reduction theory (12), high levels of uncertainty cause increases in information-seeking behavior. As uncertainty levels decline, information-seeking behavior declines (26). Meanwhile, information seeking reduces uncertainty (27). Moreover, in response to public uncertainty, the Chinese government made great effort to contain the outbreak and spread the knowledge of the disease in the media, which might help mitigate public uncertainty. Therefore, with the decline of uncertainty, decreased media use is unsurprising.

In addition, considering the result of previous study conducted in the UK during the pandemic suggesting that intolerance of uncertainty was positively associated with health anxiety (28), it is reasonable that death anxiety decreased longitudinally in the current study. In sum, meaningful changes in media use behaviors and psychological outcomes occurred during the current short-term longitudinal study, suggesting the adaptive response to the crisis in the initial phase. Differences may emerge among disaster types.

In the present study, pandemic-related media use contributes to increased death anxiety longitudinally. Previous studies in the COVID-19 pandemic also showed that more exposure to threat information from the media would increase fear of the virus and death anxiety (4, 9, 29). This might be explained by the findings from laboratory and field studies showing that threat information would elevate levels of fear (30, 31). In the pandemic, media coverage contains threat information and various death cues, thus triggers death anxiety of the public.

Sympathy for people infected with COVID-19 mediated the impact of pandemic-related media exposure on death anxiety. For the first path of the mediation process, media use was positively related to sympathy for people infected with COVID-19. Sympathy towards survivors of various disasters like terrorism, and natural disasters has been reported. In the pandemic, a study conducted in the U.S. reported sympathy toward those suffering from COVID-19 in China, and Americans when the COVID-19 began to influence the U.S (32). In laboratory settings to test the generation of sympathy, it was found that attention to target can intensify the sympathy response, and sympathy judgments were higher when made online vs. from memory (33).

Moreover, vividness is an important determinant of sympathy. Sympathy is highly attuned to visual imagery, and the more vivid that imagery is, the more likely one is to sympathize (18). Therefore, the narrative of the media, which is largely focused on emphasizing of the catastrophic nature of the pandemic, and attracted the users’ attention, caused an increasing level of sympathy towards the victims.

For the second path of the mediation process, sympathy was positively related to death anxiety. Sympathy is a complex emotion elicited by the suffering of others. It is regarded to be a great asset in disaster relief operations (34), and an essential factor contributing to the development of prosocial and moral behaviors (32, 35). However, besides the positive effect in the literature, the current study revealed the negative effect of sympathy on higher levels of death anxiety, which has received less attention.

The relationship between sympathy and death anxiety might be explained by vicarious traumatization, which may be the “cost of caring”. Previous studies have suggested that sympathy is a primitive and effortless reaction which may lead to subsequent vicarious trauma (36). Sympathy and empathy were proved to put helping professionals who worked with trauma survivors like nurses and therapists at risk of vicarious traumatization (37, 38).

In the pandemic situation, the general public was exposed to the traumatic scenes through media and developed sympathy towards the victims, putting them at risk of vicarious traumatization. Research also showed a positive association between vicarious traumatization and anxiety in the pandemic, with higher vicarious traumatization through the media predicting higher pandemic-related anxiety (22). It is plausible that sympathy for survivors leads to vicarious traumatization, further causing elevated death anxiety. This might be one reason why sympathy predicted higher death anxiety during the coronavirus pandemic. While sympathy was theorized to reflect vicarious traumatization, this construct was not directly measured in the study. Future research should incorporate validated scales (e.g., the Vicarious Trauma Scale) to empirically test this mechanism and disentangle its role in amplifying death anxiety. Our results suggested the cost of caring for others. When we sympathize with their suffering, we may also be affected by this suffering; the negative correlates of sympathy should not be ignored.

Our study didn’t support the longitudinal effect of death anxiety on sympathy for the infected. The result is not surprising. Actually, a literature review article demonstrated that death anxiety is commonly experienced by nurses and other health care workers and is associated with more negative attitudes about caring for dying patients and their families (39). Another study showed that death anxiety could predict social distance towards those infected with COVID-19 (40). Therefore, death anxiety might not predict sympathy for the infected.

Some strengths and limitations of the current study should be noted. The strengths include the longitudinal design of the study, which allows to test the directionality of the associations between media exposure and psychological outcomes. The study was conducted shortly after the declaration of the person-to-person transmission of the virus, which was in the very initial phase of the outbreak.

Limitations of the study include the relatively small sample size, and the non-representativeness of the sample, which consisted of Chinese adults. This may limit the generalizability to older populations, non-Chinese contexts, or later pandemic phases. In addition, the coefficient alpha was relatively small for the measurement of sympathy, which might be attributable to the only two items used. Researchers revealed that coefficient alpha almost always underestimates true reliability, sometimes rather substantially of a two-item scale (41). We suggest the use of more items of scale to test the research question in the future. Moreover, we did not control for contextual factors like infection/death rates during data collection. Future work should incorporate these variables to isolate media effects. We did not assess media content valence (e.g., fear-driven vs. recovery narratives), which may moderate psychological outcomes. Future studies should examine how informational tone interacts with exposure patterns. Key confounders, such as baseline mental health status, pre-pandemic media habits, and COVID-19 risk perception, were not controlled. While our focus was on temporal relationships between media use, sympathy, and death anxiety, future studies should integrate these variables to isolate causal pathways more rigorously.

More studies are needed with large sample size from all over the world to determine the generalizability of the associations between media exposure, sympathy and death anxiety found in the present study. Future studies should also examine their long-term association, as the current longitudinal study only revealed a short-term correlation. Moreover, the association can be studied in other emergency or disaster settings, such as natural disasters, as well as wars and terrorism events. Exploring the impact of media exposure and the contributing factors to death anxiety can help to develop better-tailored intervention programs to improve mental health and wellbeing.

To conclude, pandemic-related media use behaviors appeared to decrease in the initial phase of the COVID-19 pandemic, while sympathy for the infected and death anxiety were relatively stable with minor decrease. Pandemic-related media use predicted increased future sympathy for the infected, and sympathy predicted increased future death anxiety. Sympathy mediated the impact of media exposure on death anxiety. This study extends prior literature by delineating a temporal pathway from media use to death anxiety via sympathy, underscoring the role of vicarious traumatization. These findings align with crisis communication theories and highlight the dual role of media as both an informant and a stressor. These results highlight the importance of maintaining awareness of the risk of over-exposure to trauma in the media and regulating media viewing to reduce death anxiety.

## Data Availability

The raw data supporting the conclusions of this article will be made available by the authors, without undue reservation.
